# Ginkgolic acid specifically potentiates alpha 1 glycine receptors

**DOI:** 10.1186/2193-1801-4-S1-L31

**Published:** 2015-06-12

**Authors:** Galyna Maleeva, Svetlana Buldakova, Piotr Bregestovski

**Affiliations:** Bogomoletz Institute of Physiology, Ukraine, Institute de Neuroscience des Systèmes, Aix-Marseille University, France; Institute de Neuroscience des Systèmes, Aix-Marseille University, Marseille, France

**Keywords:** Glycine receptor, ginkgolic acid, patch-clamp

Ginkgo biloba extract is a neuroactive agent that is widely used for correction of age-associated impairment of memory, attention deficit and other cognitive functions. It has been shown that ginkgolides and bilobalides, Ginkgo biloba extract components, are potent blockers of glycine receptors (Kondratskaya et al., 2002, Hawthorne et al., 2006). However, the effect of ginkgolic acid, the other Ginkgo biloba extract constituent, on ligand-gated ion channels was not investigated. In the present study, using patch-clamp technique and transient transfection of different subunits in CHO cells we have shown that glycine receptors (GlyRs) are modulated by ginkgolic acid in a subunit-specific manner. After pre-application of ginkgolic acid (0.5-2 min), glycine-induced currents mediated by α1 GlyRs were strongly potentiated, while currents mediated by α2 GlyRs exhibited weak inhibition. There was no significant effect of ginkgolic acid on amplitudes of currents mediated by α3 GlyRs or on GABAARs composed of 1α/1β/2γ subunits. In order to further investigate subunit-specific effect of ginkgolic acid we have focused on possible interaction sites for this compound inside different GlyR domains. We found that mutation of three residues (T59A/A261G/A303S) in α2 subunit can convert the inhibitory action of ginkgolic acid into potentiation. Indeed, application of ginkgolic acid to cells expressing α2 T59A/A261G/A303S subunits resulted in an increase of responses to low concentrations of glycine and abolishment of the inhibitory effect, typical for wild type α2 GlyR. Our results suggest that (i) ginkgolic acid selectively enhances the function of α1 GlyRs and attenuates the function of α2 GlyRs, (ii) mutation of α2 subunit converts effect of ginkgolic acid from inhibition to potentiation.
